# Identifying Metabolic Subpopulations from Population Level Mass Spectrometry

**DOI:** 10.1371/journal.pone.0151659

**Published:** 2016-03-17

**Authors:** Christine M. DeGennaro, Yonatan Savir, Michael Springer

**Affiliations:** 1 Department of Systems Biology, Harvard Medical School, Boston, Massachusetts, 02115, United States of America; 2 Department of Physiology, Biophysics and Systems Biology, Faculty of Medicine, Technion, Haifa, 31096, Israel; The George Washington University, UNITED STATES

## Abstract

Metabolism underlies many important cellular decisions, such as the decisions to proliferate and differentiate, and defects in metabolic signaling can lead to disease and aging. In addition, metabolic heterogeneity can have biological consequences, such as differences in outcomes and drug susceptibilities in cancer and antibiotic treatments. Many approaches exist for characterizing the metabolic state of a population of cells, but technologies for measuring metabolism at the single cell level are in the preliminary stages and are limited. Here, we describe novel analysis methodologies that can be applied to established experimental methods to measure metabolic variability within a population. We use mass spectrometry to analyze amino acid composition in cells grown in a mixture of ^12^C- and ^13^C-labeled sugars; these measurements allow us to quantify the variability in sugar usage and thereby infer information about the behavior of cells within the population. The methodologies described here can be applied to a large range of metabolites and macromolecules and therefore have the potential for broad applications.

## Introduction

Many biological assays involve bulk measurements. While extremely powerful, these measurements cannot identify underlying stochastic variation and heterogeneity, which are common in many biological processes [1]. Single cell heterogeneity underscores many important biological behaviors such as bacterial persistence [2,3] and has helped to identify new subpopulations of cells [4–6]. In each case, novel techniques were required to monitor single cell variability.

Cellular metabolism determines the energetics and redox state of the cell, and proper regulation of metabolic pathways are critical for cell function. Defects in metabolism are associated with aging, neurodegeneration, obesity, diabetes, and cancer, and characterizing the cellular metabolic state in these diseases is critical for understanding and treating them (reviewed in [7–9]). Methods exist for characterizing the metabolic state of a population of cells. For example, isotopic labeling can be used in conjunction with constraint based modeling to quantify the activity of metabolic pathways. However, in some cases, such as in a tumor, there may be significant cellular heterogeneity, and characterizing this variation could be critical for treatment. While there are several methods under development that use approaches such as micropipetting, microfluidics, and cell arraying to allow single cell mass spectrometry analysis (reviewed in [10,11]), these approaches are highly specialized and technically difficult. Several recent studies have developed analytical approaches to deconvolve a mixed population using mass spectrometry measurements of metabolic incorporation of ^13^C labeled carbohydrates. One study used species specific peptides to characterize the flux of two populations within a microbial community [12]. However, this approach requires that the two subpopulations be different at the proteomic level, and precludes analysis of divergent behaviors within a genetically identical population. Another study characterized the flux and population size of two *E*. *coli* mutants with divergent metabolic behaviors by fitting flux models assuming one or two behaviors within the population [13]. However, this approach worked best with [1,2-^13^C]glucose labeling, and involved normalization of the flux of glucose into the cell, and therefore was intended for cells grown in a single carbon environment. Here, we take a similar analysis approach to determine metabolic variance in a population grown in a mixture of carbon sources, in order to address questions about metabolic choice. Our approach takes advantage of established experimental techniques and knowledge about population based mass spectrometry measurements [14,15], but extends the analysis to allow fitting of multiple subpopulations. We use a simple and easily adaptable model, which, rather than determining the flux within in the pathway, can easily identify divergent uptake behaviors. This model works well with [U-13]C labeled carbohydrates, which maximizes opportunity to identify metabolic mixing and reflux [16]. We validate and characterize this analysis using a variety of experimental, rather than simulated, data to ensure the robustness of our approach. For all these reasons, this approach is simple to perform and transferrable to many biological systems.

**Fig 1:**
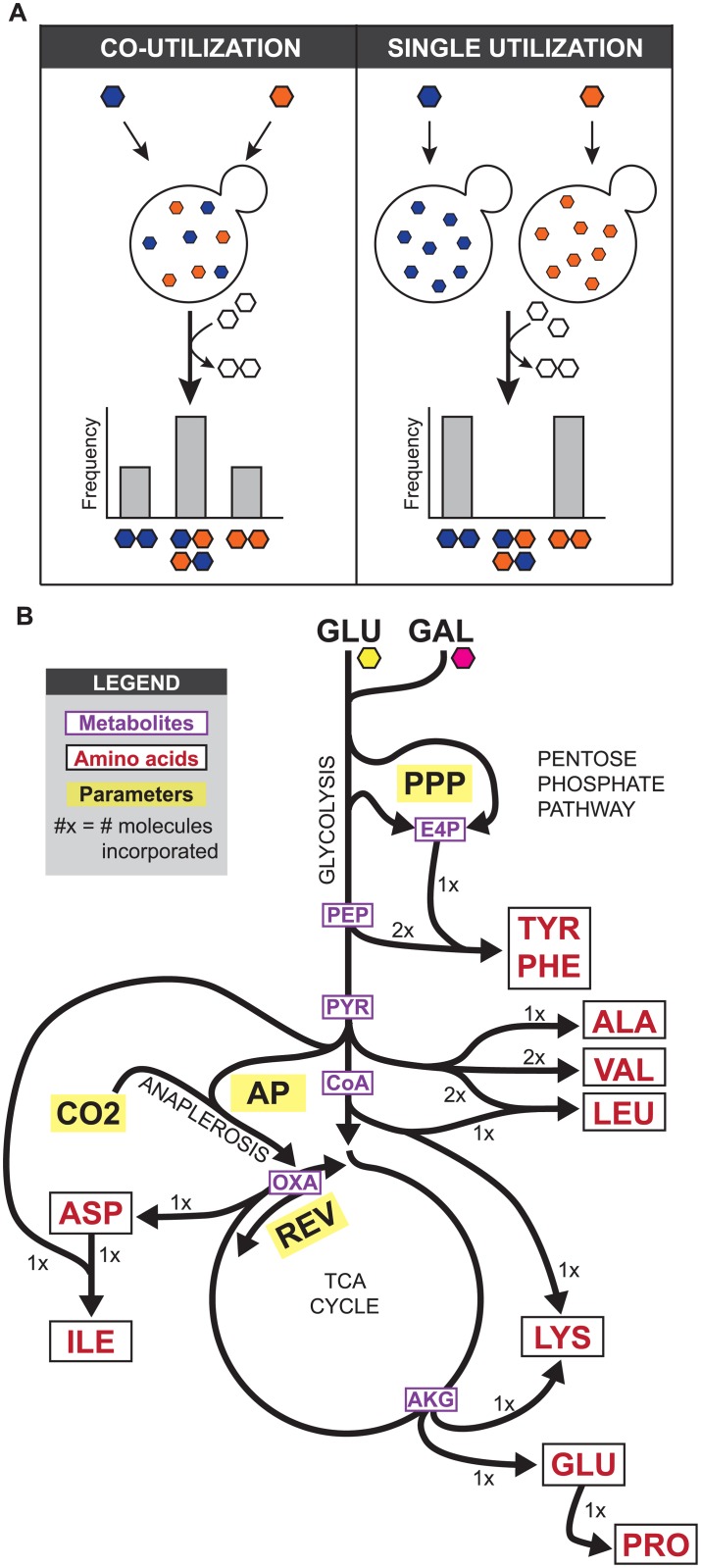
Metabolic variance can be inferred from macromolecule composition. (A) In any system in which a cell can uptake two different metabolites (blue and orange hexagons) and use them interchangeably to build a macromolecule, composition of the macromolecule can provide information about metabolic variance. If cells are co-utilizing both metabolites (left) there will be macromolecules composed of a mixture, while if there are subpopulations of single utilizing cells (right), there will be only pure macromolecules. (B) A model for amino acid biosynthesis from glucose (GLU) and galactose (GAL) in yeast, based on a simplified version of central carbon metabolism. Amino acids are shown in red, metabolites are shown in purple, and free biochemical parameters are highlighted in yellow. Numbers indicate the number of each metabolite incorporated into the amino acid.

Assume a population of cells consumes two sugars from the media concurrently; is it possible to determine if all cells use both sugars, or if some cells use one while others use the other? These two scenarios are depicted schematically in Fig 1A. To date, this question has been difficult to address, but our novel analysis approach can infer the answer. In the simplest scenario, a cell is grown in the presence of two metabolites (i.e. glucose and galactose) that can be used interchangeably to create a macromolecule (i.e. an amino acid). If the entire population co-utilizes both of the metabolites, the resulting macromolecule can be composed either from two of the same metabolite or one of each (Fig 1A, left panel). Alternatively, if there are two subpopulations of cells which each use only one of the two metabolites, the population as a whole may uptake the same amounts of each metabolite as in the first scenario, but the distribution of the metabolite in the macromolecule will be different; all macromolecules will be composed entirely of one or the other metabolite (Fig 1A, right panel). Because most current models determine metabolic flux by fitting to a model that assumes a single population behavior, the scenarios above are indistinguishable. However, if the data were instead fit to a model that allows multiple populations, these two scenarios are distinguishable. Here, using mass spectrometry analysis of amino acid composition of yeast in a mixed sugar environment, we demonstrate that we can distinguish between co-utilization and single utilization of two subpopulations. Using proof of principle experiments, we find that this data provides sufficient information to make predictions about whether there is a subpopulation, and the relative subpopulation size and sugar utilization ratio. We then apply this approach to a biological question to demonstrate that a wild yeast strain grown in a mixed glucose and galactose environment co-utilizes the two sugars.

## Results

### An Amino Acid Biosynthetic Model Recapitulates the Different Behavior of One- and Two-State Populations

**Fig 2:**
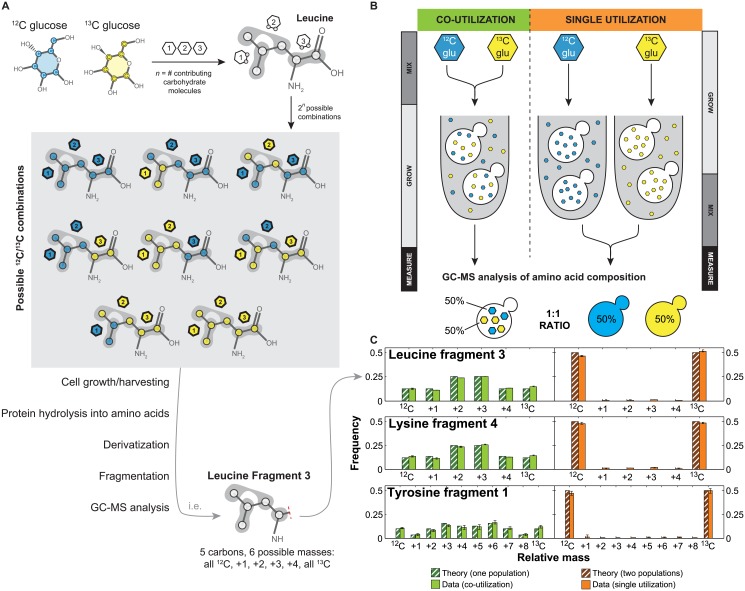
Amino acid composition reflects sugar utilization. (A) Differentially labeled ^12^C and ^13^C glucose are incorporated into amino acids; for example, leucine contains contributions from three different carbohydrate molecules, creating 2^3^, or 8, possible ^12^C/^13^C combinations. After derivatization, this creates amino acid fragments with carbohydrate compositions predictable by our model. (B) Proof of principle co-utilization experiment created by mixing ^12^C and ^13^C glucose and growing cells in the mixture (“mix then grow”) and single utilization experiment created by growing cells separately in each carbon source and combining them (“grow then mix”). (C) The amino acid metabolic model recapitulates amino acid composition data from GC-MS analysis of these experiments, with co-utilization closely matching the one-state model and single utilization of subpopulations closely matching the two-state model (Pearson’s correlation is 0.997 and 0.967, respectively, over all fragments). For the data, bars represent the mean of three biological replicates, with error bars corresponding to the standard error of the mean. For the theory, the following biochemical parameters were used to calculate the distributions: CO2 = 0.1, AP = 0.9, REV = 0.5, PPP = 0.5.

We used amino acid composition as a read-out of sugar utilization in yeast, as amino acids are built from multiple metabolic precursors derived from sugars like glucose and galactose. As we were mainly interested in differences in uptake, we simplified current models of metabolism [17], taking into account only the major pathways responsible for amino acid synthesis from glucose and galactose, including glycolysis, anaplerotic reactions, and the tricarboxylic acid (TCA) cycle (Fig 1B). Additionally we fit the relative flux at each of the following metabolic branch points: anaplerotic synthesis versus TCA cycle (AP), the reverse vs forward TCA cycle (REV), the composition of erythose-4-phosphate produced by the oxidative and non-oxidative pentose phosphate pathway (PPP), and the fraction of carbon dioxide incorporated from the air versus recaptured from glycolysis (CO2) (Fig 1B). We modeled all isotopic species, and then calculated cumomer distributions for each of the amino acid fragments. This model then allowed us to predict the composition of each amino acid given single or co-utilization under specific biochemical conditions; for example, leucine is composed of carbons derived from three metabolites (an acetyl CoA and two pyruvates) derived from independent carbohydrate molecules, producing 2^3^, or 8, possible isotopomers (Fig 2A). Cultures were grown in a mixture of heavy and light sugars; for all experiments, we used [U-13C] glucose as the heavy sugar, as it is optimal for differentiation of subpopulations. After harvesting, cells were processed as previously described [17]. Briefly, the proteins were acid hydrolyzed into amino acids, which were then derivatized with N-tertbutyldimethylsilyl-N-methyltrifluoroacetamide and measured by GC-MS. Previous work has shown this can produce 5 distinctly measurable fragment of most amino acids [18]; our model can predict the theoretical mass distribution of each of these fragments in the case of co-utilization and single utilization.

In order to validate our method, we performed a proof-of-principle experiment where we grew cells in scenarios where they co-utilized or single utilized carbon sources. To achieve this, we either grew cells in a 50%-50% mixture of ^12^C and ^13^C glucose (experiment referred to as “mix then grow”) or grew them independently in either 100% ^12^C or ^13^C glucose and then mixed them in a 50%-50% ratio just before cell lysis (experiment referred to as “grow then mix”) (Fig 2B). The ^12^C and ^13^C glucose are biologically equivalent and should cleanly be co-utilized and single utilized, respectively.

Using our model, we calculated the expected amino acid distributions for single and co-utilization in glucose (Fig 2C). GC-MS analysis of the experimental samples showed that the data closely matched our theory for the appropriate number of subpopulations (Fig 2C). For the 29 amino acid fragments that we will analyze here (S1 Table), the overall Pearson correlation for the mix then grow (one-state model) experiment is 0.97 for the one-state theory and the grow then mix (two-state model) experiment is 1.00 for two-state. When using the wrong model (i.e. the two-state model to fit the one-state experiment or one-state model to fit the two-state experiment) the correlation coefficient drops to 0.33 and 0.35 respectively, which is similar to that of the one- and two-state theories to each other (0.33). The remaining correlation is partly due to the fact that fragments produced from a single carbon source (i.e. fragment 5) have the same distribution in both models; removing these fragments decreases the correlation coefficient to 0.13 and 0.15 for the wrong models, and the model-to-model correlation to 0.14. However, the coefficient for the correct models, even without these fragments, remains 0.96 and 1.00. These results indicate, first, that one- and two-state populations are distinguishable by this method, and second, that our model accurately recapitulates the observed amino acid distributions.

### Fitting GC-MS Data with One- and Two-State Models Allows Inference of Subpopulation Characteristics

**Fig 3:**
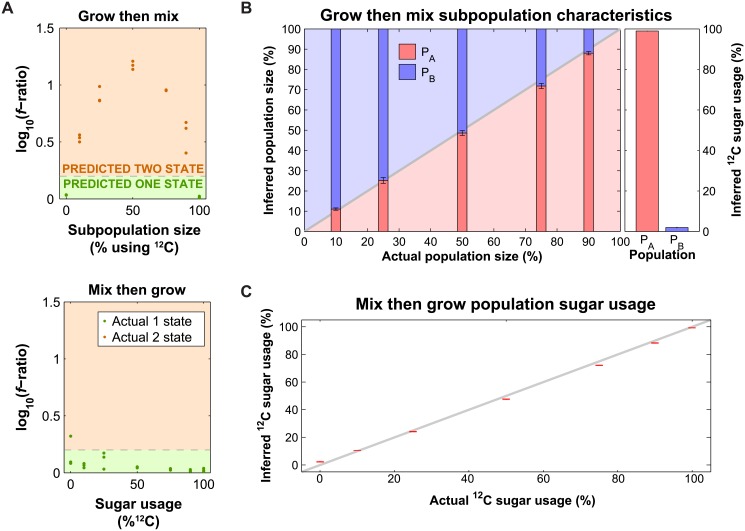
Fitting of amino acid composition with one and two-state models can predict population behavior. (A) Prediction of the number of states in the population for the “grow then mix” (top) and “mix then grow” (bottom) data, based on the fit of the one and two-state models. The gray dashed line indicates the threshold where the log_10_ of the ratio of *f*_*one-state*_ to *f*_*two-state*_ is 0.2; a population is predicted to be one-state below this threshold (green) and two-state above this threshold (orange). Color of the points indicates whether the population is actually one- or two-states (green and orange, respectively). (B) Inferred subpopulation size (left) and sugar usage (right) based on fitting of the “grow then mix” data. Bars represent the mean of three replicates, with error bars corresponding to the standard error of the mean. (C) Sugar utilization in the “mix then grow” (co-utilization) experiment determined by fitting the data with a one-state model, compared to its actual sugar usage. Error bars represent the standard error of the mean (*n* = 3).

Given the success of our model at simulating the experimental data, we asked if fitting our model to the amino acid data could predict (1) if subpopulations are present, (2) the relative size of any subpopulations, and (3) the sugar usage fraction of each subpopulation (in this case, the fraction of ^13^C-glucose and ^12^C-glucose used). To evaluate the models and determine whether subpopulations are present, we calculate the square root of the sum squared of the residuals between the measurement and theory (*f*). Because the two-state model has more parameters than the one-state model (11 vs. 5), it always has a lower *f* value than the one-state model even if the one-state model is correct, due to overfitting. We found that neither the Akaike information criterion [19] nor Bayesian information criterion [20] discriminated between the models, likely due to the relatively low number of total measurements compared to the number of parameters. We therefore chose an empiric threshold of 0.2 for the log difference of *f* as a cut-off to distinguish between one- and two-state models (Fig 3A, bottom panel). Above this threshold, the two-state model is determined to fit best; below this threshold, a one-state model is determined to fit best. This threshold corresponds approximately to a 5% false discovery rate (20/21 experiments classified as two-state), but given the limited number of samples it is not possible to rigorously define the threshold in this way.

Using this threshold defined by analysis of the one-state model, we tested whether this threshold was able to accurately classify two-state models. We created 15 two-state “grow then mix” experiments by growing cultures in pure ^12^C- or pure ^13^C-glucose and pooling in varying ratios (Fig 2B). Additionally, we included 6 new samples grown either in pure ^12^C or ^13^C as controls, allowing us to test our predictions. We found that this threshold correctly predicted the number of states in all 21 of the one and two-state population samples; as expected the difference in *f*-ratio is maximized when the ^12^C or ^13^C glucose samples are mixed 50:50% (Fig 3A, top panel). Additionally, we find that the two-state model accurately predicts both the fraction of cells (average absolute deviation of 1.9 ± 1.3%; error is one standard deviation) and the sugar usage percentage (average absolute deviation of 1.5 ± 0.7%) of each subpopulation (Fig 3B). These results show that our analysis can separate between single and co-utilization.

### Limitations on Subpopulation Characterization

We have shown that we can predict the presence, size and sugar utilization of subpopulations when they are each exclusively using different sugars, but we wanted to determine whether we could distinguish two subpopulations of cells that are both co-utilizing, but at different ratios, while still accurately predicting the subpopulation size and sugar utilization. To test this, we first prepared “mix then grow” cultures in triplicate in varying ratios of ^12^C- and ^13^C-glucose and fit the resultant amino acid distributions with one- and two-state models. We observed that the predicted sugar usage in the one-state model correlates closely with the known experimental ratio (average absolute deviation = 1.7 ± 0.94%, error is one standard deviation) (Fig 3C).

**Fig 4:**
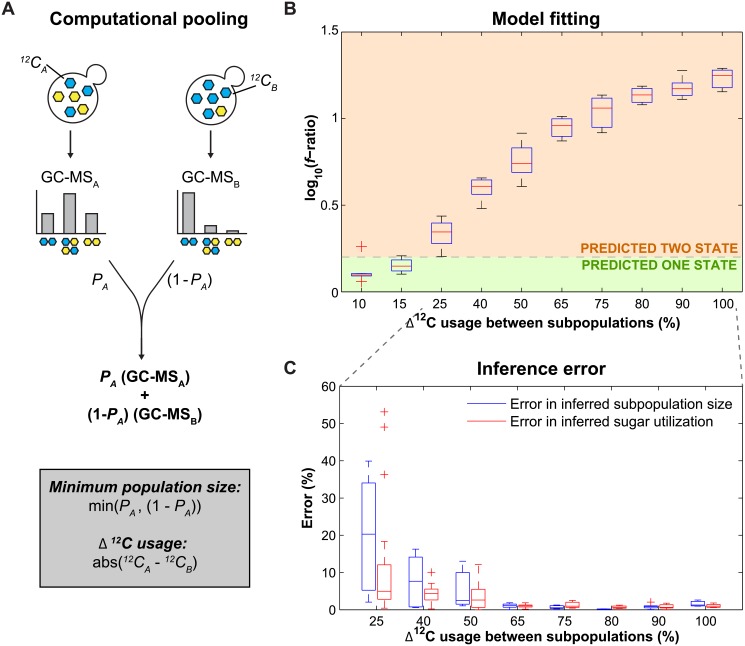
The difference in sugar utilization between two subpopulations is the major determinant of inference error. (A) Computational pooling was performed by summing two sets of experimental GC-MS amino acid distributions (GC-MS_A/B_) with different ^12^C usage fractions (^*12*^*C*_*A/B*_), weighted by the desired population ratio (*P*_*A*_ and 1-*P*_*A*_). The minimum population size (min(*P*_*A*_,(1-*P*_*A*_))) and difference in ^12^C usage between subpopulations (abs(^*12*^*C*_*A*_-^*12*^*C*_*B*_)) were determined for each computational pool. (B) Inferred subpopulation number based on the log_10_ *f*-ratio of the computationally pooled data, with dashed line indicating the 0.2 threshold. Boxes represent the interquartile range with a red line at the median, and outliers >1.5x the interquartile range are shown as a red plus sign. (C) Inference error in subpopulation size (blue) and sugar usage (red) from computationally pooled data that fit best with a two-state model. Boxes represent the interquartile range with a line at the median, and outliers (>1.5x the interquartile range) are shown as a plus sign.

To expand the number of mixing ratios and relative sugar utilization ratios for analysis without having to perform combinatorially large numbers of experiments, we computationally weighted and combined pairs of experimental data to "simulate" different pooling ratios (Fig 4A). To check that computationally pooled data was equivalent to experimental data, we pooled the data from pure ^12^C- and ^13^C-glucose samples in the same ratios as the “grow then mix” experiment and compared them to the equivalent experimental result. The raw data from the experimental pooling was statistically indistinguishable from the computational pools (S1A Fig). Fitting of these computationally pooled distributions (S1B and S1C Fig) produced results similar to the experimental data (Fig 3B), with an average absolute deviation of 0.87 ± 0.61% for population size and 1.6 ± 1.0% for sugar utilization.

Given the consistency of the results, we expanded our computational pooling approach to test the ability to accurately infer varying the sugar utilization of each subpopulation. To do this we pooled the one-state experimental data (Fig 3B) in a 50–50% ratio. We found that under these conditions, the two-state model can predict the size (average absolute deviation = 7.0 ± 11%) and sugar utilization (average absolute deviation = 4.2 ± 8.2%) of subpopulations with at least a 25% difference in sugar utilization (Fig 4B). The larger the difference in sugar utilization, the higher the accuracy of the predictions; for example, subpopulations with at least a 40% difference had average absolute deviation of 2.9 ± 4.4% and 2.1 ± 2.4% in size and overall sugar usage (including both subpopulations), respectively (Fig 4C).

In order to more completely determine the limitations in separating between one and two populations we tested our ability to infer relative subpopulation size and the sugar utilization ratios when both are varied. In order to characterize the limitations of these two characteristics within the entire space, we calculated the average absolute deviation of the inferred subpopulation size and sugar utilization while varying the thresholds (S2A Fig). When the difference in sugar utilization was at least 25%, the average absolute deviation was 5.5 ± 7.8% for inferred subpopulation size and 7.8 ± 16% for inferred sugar usage; when the difference in sugar utilization was at least 40%, those statistics dropped to 3.2 ± 5.6% and 4.1 ± 9.0%, respectively. Hence, the primary limitation in separating between one- and two-state models, as well as accurately inferring subpopulation size and sugar utilization is the difference in sugar utilization between the two subpopulations, with the subpopulation size playing a minor role (S2A Fig). The limitations calculated in this section are sensitive to the threshold chosen; one can increase the accuracy of the inferred fits by raising the *f-*ratio threshold, thereby increasing the stringency of calling something a two-state population (S2B Fig). This approach will produce more false negatives (one-state predictions when a two-state model is correct), but provide more confidence in the inference in populations identified as two-state. Depending on the application and the intended follow-up, a more or less stringent threshold may be beneficial.

### Inferring Subpopulation Characteristics after a Sugar Shift

**Fig 5:**
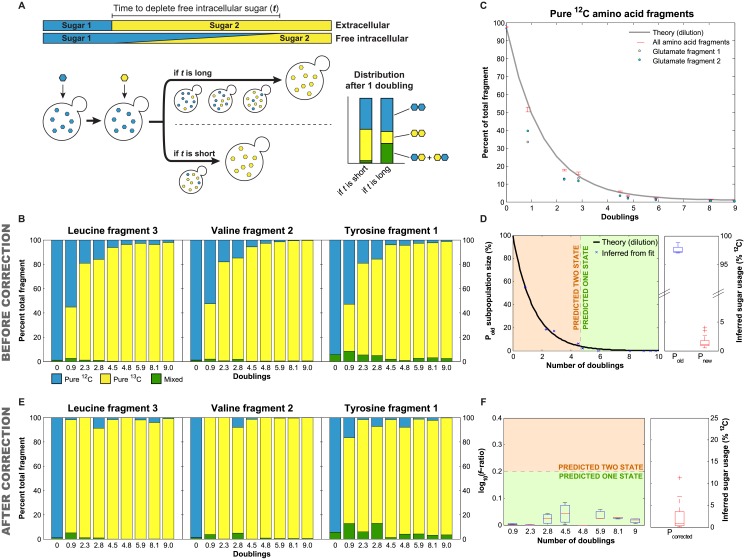
Switching between carbohydrate sources causes minimal production of intermediate mass amino acids. (A) When cells are subjected to a shift in carbon sources, production of intermediate mass amino acids (indicated by green bar) depends on the time scale of depletion of free intracellular sugar. (B) The relative mass distribution of three amino acid fragments over 9 doublings after a carbon source switch. Pure ^12^C species are shown in blue, pure ^13^C species in yellow and mixed, intermediate mass species in green. Bars represent the mean of two biological replicates. (C) The mean percent of pure ^12^C amino acid fragments, corresponding to the blue species in (B). Red error bars represent the standard error of the mean over all 29 amino acid fragments. The mean for each fragment was calculated from two biological replicates. The gray line represents decay of the pure ^12^C species by growth rate mediated dilution. Points represent the mean of glutamate fragments 1 and 2 (yellow and green, respectively). (D) Time course data was fit with both one and two state models, and the inferred subpopulation size (left) and sugar utilization (right) were determined. Subpopulation size of P_old_ is shown by blue x’s, with error bars corresponding to the range (*n* = 2), and dilution by growth rate shown by the black line. Sugar utilization of P_old_ and P_new_ over all time points is shown in blue and red, respectively, with the box representing the interquartile range and a line indicating the median value. (E) The relative mass distribution of three amino acid fragments over 9 doublings after a carbon source switch after correction for dilution, plotted as described in (B). (F) Fitting of the corrected data with one- and two-state models allowed inference of the number of subpopulations (left) and sugar utilization (right). Boxes represent the interquartile range with a line at the median, and outliers >1.5x the interquartile range are shown as a plus sign.

The experiments described up to this point were conducted at steady state. However, depending on the biological and technical details of an experiment, it is not always possible to grow cells to steady state. Our approach has two potential complication when monitoring a population in a dynamic environment. First, when switching carbon sources, how long does it take the population to reach steady state? If there is a significant time before the composition of new synthesized macromolecules reflect current usage (e.g. because of internal stores) this will contribute to our inference. Second, if a population of cells is switching back and forth between carbon utilization strategies, macromolecule composition could appear similar to co-utilization. Both of the scenarios share the same limitation, namely, the time scale it takes for the internal composition to reflect current usage of metabolites. One can measure this by shifting cells from ^12^C to ^13^C glucose. If there are no internal stores or recycling, amino acids will be composed of only pure ^12^C and ^13^C amino acids, with no intermediate mass species. For example, in a shift from a ^12^C to a ^13^C carbon source, there will be a population of light ^12^C amino acids that were synthesized before the shift (P_old_) and heavy ^13^C amino acids synthesized afterwards (P_new_), but no mixed ^12^C/^13^C amino acids. Conversely if there is a significant contribution of internal stores and recycling, mixed ^12^C/^13^C amino acids will be generated for a considerable time after the shift. Our model cannot distinguish between intermediate mass species from internal stores and those from co-utilization, so this could make transition period measurements inaccurate (Fig 5A).

To determine the magnitude of the internal stores, we switched cells from ^12^C-glucose to ^13^C-glucose, and monitored the amino acid composition for the subsequent 9 doublings. To more accurately measure the amount of new amino acids made from pure ^13^C versus ^12^C/^13^C mixture, we used a subset of 8 amino acid fragments (S1 Table) that are not produced by anaplerotic synthesis (incorporating CO_2_ from the air), did not have interfering species, and were synthesized from multiple carbohydrate sources. The maximum amount of intermediate mass species (the sum of all species not purely ^12^C or ^13^C in composition) in these fragments range from 0.9% to 5.7%, with a mean of 2.3%, indicating that the pool of free intracellular sugar is quickly depleted (Fig 5B), consistent with previous studies [21]. This maximum concentration of intermediate mass species occurs at our first measured experimental point after the media switch (0.9 doublings). After this first time point, we observe that the pool of ^12^C/^13^C amino acids decays exponentially as would be expected from dilution by growth rate with no new synthesis (Fig 5C). In total, we found that cells convert to using pure ^13^C in less than 1 doubling.

We fit this data with our one and two-state models. Because this is a kinetic, rather than a steady state, experiment, the variability in the amino acids identified here arises from a temporal change (before and after the sugar shift), rather than differences between cells. Our beginning time point is at steady-state (~100% ^12^C-amino acids) and at the end of our assay we have reached a new steady-state (~100% ^13^C-amino acids). When the cells have doubled once, half the amino acids should be ^12^C and half ^13^C. As expected, after a small number of doublings, we find that the two-state model fits best. The model infers both the old, ^12^C population of amino acids (P_old_) and the new, ^13^C population (P_new_) for 4.5 doublings, at which point P_old_ represents only 2^−4.5^, or ~5% of the overall pool of amino acids. After growth rate mediated dilution causes P_old_ to drop below 5%, it is no longer detectable by our method and a single population model best fits our data. All the fits accurately infer both the relative population sizes (average absolute deviation = 1.2 ± 1.4%) and the ^12^C/^13^C composition of the amino acids in both populations (average absolute deviation = 1.9 ± 1.2%) (Fig 5D). This supports the prediction that the recycling rate of the internal amino acid stores is not the same order of magnitude as import.

Given that P_old_ decays predictably according to growth rate, it should be possible to correct for this population and fit P_new_ to a single or two state model, even at the initial time points. While this is a rough correction, if it works well, the best fitting model should be one in which all new amino acids are made from ^13^C. To test this, we corrected each time point by subtracting out the amino acid distributions from the previous time point, weighted by the amount of dilution (2^-doublings^), and dividing by the total to renormalize to 1. After correction, the amino acid fragments are composed almost entirely of ^13^C, even at 0.9 doublings (Fig 5E). Fitting the corrected data predicts a single population (P_corrected_) using > 95% ^13^C at all time points (Fig 5F), reflecting only the sugar usage after the switch (P_new_). This demonstrates that this method can correctly infer the behavior of a population, even shortly after a sugar shift.

It is possible that switching between nutrients could elicit higher rates of metabolite recycling. To test this we switched the carbon source (^13^C-glucose to ^12^C-galactose and ^12^C-raffinose to ^13^C-glucose). Like the switch from ^12^C-glucose to ^13^C-glucose, the pre-switch carbon source decays by growth rate dilution and the population achieves rapid steady state with the presence of minimal mass intermediates (S3 Fig); using the same metric as above, we observed an overall mean of 3.6% intermediate mass species across all of the carbon shifts. Therefore, amino acid and metabolite recycling does not substantially contribute to the metabolite pool for new amino acid synthesis (Fig 5C, S3 Fig). In contrast, we observe that glutamate fragments decay at a rate greater than predicted by doubling alone (Fig 5C). This difference is expected as glutamate is converted into proline and therefore has an effective decay rate that is larger than dilution alone. Overall, these experiments show that there are minimal limitations on this analysis after nutrient switching.

### A Wild Yeast Strain Co-Utilizes Glucose and Galactose

**Fig 6:**
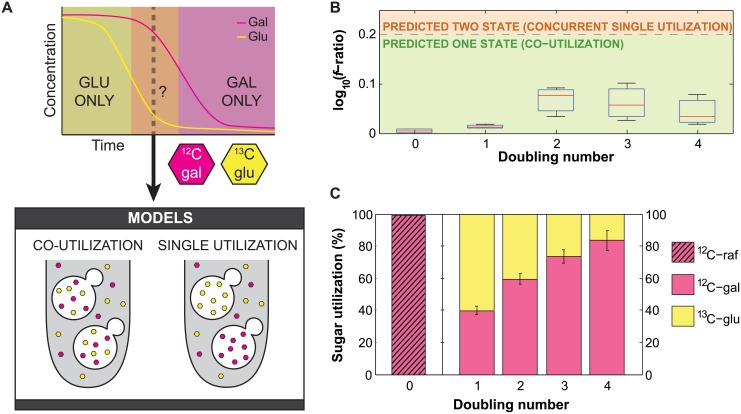
The wild yeast strain, BC187, can co-utilize glucose and galactose. (A) In order to distinguish co-utilization and single utilization of glucose and galactose, cultures were grown at steady state in a sugar concentration regime that leads to depletion of both glucose and galactose from the media at roughly equal rates (2% ^12^C galactose and 0.022% ^13^C glucose). (B) Inferred subpopulation number after correction for dilution from cell growth (*n* = 3). Subpopulation inference is based on the log_10_ *f*-ratio of the data, shown by boxes representing the interquartile range with a line at the median. The dashed gray line indicates the 0.2 threshold; below this (green), populations are predicted to be one-state, indicating co-utilization, and above (orange) two-states are predicted. (C) Inferred sugar utilization of the population, after correction for dilution from cell growth. Mean ^12^C-raffinose (pink with black diagonal lines), ^12^C-galactose (pink), and ^13^C-glucose (yellow) utilization are represented by bars (*n* = 3), with error bars corresponding to the standard error of the mean. Data reflects the glucose and galactose utilization specifically during the current doubling, after correction for previous species.

In a model system for studying co-utilization, populations of the budding yeast, *Saccharomyces cerevisiae*, simultaneously deplete glucose and galactose from media [22]. While this could be due to co-utilization, another study suggested that this depletion could be attributed to noise in the assay or variability in single cell behavior, proposing instead that the sugars were used sequentially [23]. In order to separate between these two possibilities, we grew a culture of the wild yeast strain BC187 at a steady state concentration of glucose and galactose known to cause roughly equal depletion of both sugars from the medium (Fig 6A). Briefly, we shifted a culture from pre-growth in ^12^C-raffinose to medium containing 0.022% glucose and 2% galactose and measured the amino acid composition over four doublings.

We then fit this data with our model to infer if cells are co-utilizing glucose and galactose. As in the previous kinetic experiment, we expected to observe a population of old, ^12^C amino acids from growth in raffinose and new amino acids of an unknown isotopic mixture from growth after the shift. To correct for this old population, we calculated the expected dilution due to growth rate as above and subtracted this fraction from the isotopic distribution for each amino acid fragment. This correction was done between each consecutive pair of time points because the cells induce genes that can affect the relative uptake rate of glucose and galactose. We then fit the remaining population of amino acids, allowing us to detect whether the new population of amino acids is generated by co-utilization or single utilization. If different cells were using each sugar, or if the sugars were used sequentially, we would expect a two-state model to fit the data. However, we found that a one-state model fit the data best at all time points, supporting co-utilization of glucose and galactose (Fig 6B). At 1 doubling, the culture is using 40% ^12^C-galactose and 60% ^13^C-glucose ± 3% (Fig 6C). The utilization shifts gradually over time, reaching 84% galactose and 16% glucose ± 6% by 4 doublings. Given the low density of cells in the experiment, this is probably due to induction of galactose transporters [24]. These results support our previous assertion that yeast strains are able to co-utilize glucose and galactose in a mixed sugar environment [22].

## Discussion

Here we present a novel analysis method for mass spectrometry data that allows the detection of metabolic variation within a population. This method builds off of standard metabolomics methodologies but uses a constraint-based model that allows for the existence of two subpopulations of cells. We show in a number of proof of principle experiments that this methodology is able to distinguish between a population co-utilizing two carbon sources and two subpopulations with different carbon utilization characteristics, over a wide range of sugar utilization strategies and relative population sizes. Specifically, as implemented here, our approach can infer the relative size and sugar utilization of each subpopulation, with an average absolute deviation of less than 8%, as long as the difference in utilization of the heavy and light sugar is at least 25% between the two subpopulations.

Additionally, our proof of principle experiments support or extend several observations. Previous work has shown that free intracellular metabolites are rapidly turned over. Other studies had reported a second, less labile pool of amino acids stored in the yeast vacuole [25]. By measuring the amount of intermediate mass isotopic species after a nutrient shift, we show that the vast majority of new amino acids are made from newly imported sugars rather than internal stores. Furthermore, after a media switch, the decay of most old amino acids was exponential, suggesting that amino acid turnover does not contribute substantially to synthesis of new amino acids. The exceptions to this are glutamate and arginine, which both decay more rapidly than expected by dilution alone, which is expected, as these amino acids or their interconvertible species are used as precursors for proline, isoleucine, methionine, threonine, arginine, and lysine synthesis.

We then used this approach to resolve an outstanding biological question—do yeast co-utilize glucose and galactose in a mixed sugar environment? Several lines of evidence have suggested that it is possible for isogenic populations to have a bistable metabolism [26,27] or bistable expression of sugar utilization systems [28,29]. Additionally, a recent study found that there is isogenic variability in the response time of the galactose utilization genes, leading to the argument that co-utilization in individual cells was minimal [23]. Instead, we found here that yeast do co-utilize glucose and galactose. In our growth conditions, there was no evidence of a subpopulation of cells with a distinct sugar utilization strategy.

In this work we used a simplified model of carbon metabolism, which was sufficient to achieve our initial biological goal of detecting heterogeneity in sugar utilization strategies. In the future, this work could be extended in a number of ways that would broaden its applicability. A more extensive model could be used to allow measurement of metabolic variability. This analysis would be aided by choosing isotopic media components that are optimized for differentiating between choices in metabolic flux [18]. Our current model only incorporates isotopic data from amino acids, but this could be extended to any other metabolite that can be synthesized from multiple metabolic precursors. These extensions of the system will be critical for applications in human cells, where many amino acids are essential. Our current model also ignores the potential for cross-feeding between cells but this could be included in scenarios where it is relevant [30,31]; in our experiments we believe cross-feeding to be negligible given the similarity in profiles of all amino acids and the greatly reduced rates of growth in yeast strains forced to cross-feed [32]. Finally, our model assumed up to two discrete underlying metabolic states. The analysis could be extended to determine the most likely distribution of metabolic states that account for a given set of isotopic measurements.

While this method is not a single cell method, it is still able to reveal a wealth of information about variability in cellular metabolism with direct implications for behavior at the single cell level. It will be extremely interesting to see if we detect large amounts of heterogeneity as we use this technique to measure normal or cancerous human tissue. While useful in its own right, we believe one of the great benefits of this method is that these analysis concepts can be easily translated to other systems. For example, proteins are made from multiple amino acids, so a method similar to the one described here should allow one to determine whether cells with different metabolic profiles have different proteomic profiles. We believe the basic strategy provided will be creatively used by others in the community to infer information that was previously unmeasurable.

## Methods

### Strains, Media, and Culture Conditions

Prototrophic *Saccharomyces cerevisiae* strains were grown at 30°C in synthetic media (1.45 g yeast nitrogen base without amino acids or ammonium sulfate and 5 g ammonium sulfate per liter) supplemented with either ^12^C-glucose, ^12^C-galactose, ^12^C-raffinose or ^13^C_6_-glucose (99% purity from Cambridge Isotope; CLM-1396-PK). All strains were S288c, except for the wild BC187 strain. Cells were harvested by spinning for 3 minutes at 3000xg, and cell pellets were stored at -80°C until processing for mass spectrometry.

### Grow then Mix and Mix then Grow Experiments

Cells were grown in triplicate to saturation in synthetic media supplemented with ^12^C-glucose. Cells were then diluted 1:50 into the fresh media and grown for 4 hours to reach mid-log. Each replicate was diluted 1:10 using synthetic media with no carbon source, washed three times with synthetic media with no carbon source and grown for 16 hours at 30°C in 1mL of synthetic media. For the mix then grow experiment, this media contained the following ratios of ^12^C-glucose to ^13^C-glucose: 1:0 1:9, 1:3, 1:1, 3:1, 9:1, or 0:1. For the grow then mix experiment, this media was supplemented with either ^12^C-glucose or ^13^C-glucose, and these cultures were pooled after growth at ratios of 1:0 1:9, 1:3, 1:1, 3:1, 9:1, and 0:1.

### Switch and Dynamics Experiments

Cells were grown to saturation in synthetic media supplemented with ^12^C-glucose. Cells were then diluted 1:50 into the same media, grown for 4 hours to reach mid-log, washed three times with synthetic media with no carbon source and resuspended in synthetic media with ^13^C-glucose to an OD600 of 0.195. This sample was serially diluted two-fold in synthetic media with ^13^C-glucose, resulting in 10 samples with different initial densities. The OD_600_ of each sample was measured before harvesting and was used to calculate the number of doublings. For the sugar shift experiments, cells were grown to saturation in media containing the initial carbon source, diluted back 1:10,000 and grown to saturation again in the initial media. Cells were then diluted 1:50 into the initial media, grown for 4 hours to reach mid-log, washed 3 times in media lacking a carbon source and resuspended in the media containing the second carbon source. For the glucose/galactose co-utilization experiment, a BC187 strain was pre-grown in YPD, diluted back into synthetic media + 2% ^12^C-raffinose and grown to early mid-log, washed three times in synthetic media without sugar and resuspended in synthetic media with 2% ^12^C-galactose and 0.022% ^13^C-glucose.

### GC-MS Analysis

Frozen cell pellets were prepared for mass spectrometry analysis as previously described [33]. Briefly, pellets were washed once in 0.9% sodium chloride, resuspended in 6N hydrochloric acid and incubated at 105°C for 18–24 hours. Tubes were then opened and incubated at 95°C for 24 hours to dry the hydrolysate. The hydrolysate was derivatized with N-tert-Butyldimethylsilyl-N-methyltrifluoroacetamide with 1% tert-Butyldimethylchlorosilane (Sigma 375934) in dimethylformamide (Sigma 227056) at 85°C for 1 hour and transferred to a GC-MS vial. A GC-MS standard with equimolar amounts of all amino acids was prepared and derivatized as well. Samples were analyzed on a Waters Quattro micro GC/MS/MS with an Agilent 6890 GC and CTC CombiPAL autosampler as previously described [33].

### Processing of GC-MS Data

The resulting data was extracted and processed used FiatFlux [33], with one modification. FiatFlux calculates the distribution of ^12^C and ^13^C isotope by deconvolution; instead, we calculated the expected observed isotopic distribution for each specific isotopic species (this is a distribution due to the fact that neither the ^12^C or ^13^C carbohydrates are completely pure) and determined the weighting of each specific isotopic species that best fit the data. Raw amino acid fragment mass distributions derived from FiatFlux are documented in S2 Table. The modified code is available on the Dryad Digital Repository (doi:10.5061/dryad.gf80t).

### Fitting the GC-MS Data

A simplified model of amino acid biosynthesis was developed as schematized in Fig 1A, accounting for glycolysis, anaplerosis, the TCA cycle, and parts of the pentose phosphate pathway (S1 Text). Normalized amino acid distributions were fit to the one-state (5 free parameters) and two-state (11 free parameters) models in MATLAB using the fmincon function. For fitting, S1 Table identifies the 29 amino acid fragments used for fitting, as well as the subset of 8 fragments used to analyze the mass intermediates after a carbon source shift in Fig 4 and S3 Fig. Fit was evaluated by square root of the sum squared of the residuals between the measurement and theory, omitting single outliers at both ends of the distribution. The best model was selected using a threshold of log_10_ (*f*_*one-state*_ / *f*_*two-state*_) of 0.2, above which the population was considered to be two-state. Fitting data from experimental and computational data, including all biochemical parameters are listed in S3 and S4 Tables. The code is available on the Dryad Digital Repository (doi:10.5061/dryad.gf80t).

## Supporting Information

S1 Fig Analysis of computationally pooled data produces equivalent results to analysis of experimental data. (A) Amino acid distributions are equivalent in the experimental and computationally pooled data. The relative percent of each mass species in the 29 amino acid fragments used for this analysis in the experimental and computationally pooled data (3696 total, across 3 biological replicates) is plotted as a blue x. The gray line represents x = y, and Pearson’s correlation was used to calculate *r*. (B) Prediction of the number of states in the population based on the fit of the one and two-state models. The gray dashed line indicates the threshold where the log_10_ of the ratio of *f*_*one-state*_ to *f*_*two-state*_ is 0.2; a population is predicted to be one-state below this threshold (green) and two-state above this threshold (orange). The color of the circles indicates whether the population is actually one- or two-states (green and orange, respectively). (C) Inferred subpopulation size (left) and sugar usage (right) based on fitting of the pooled data. Bars represent the mean of three replicates, with error bars corresponding to the standard error of the mean.
(EPS)

S2 Fig Analysis of the effects of relative subpopulation size, sugar utilization, and *f-*ratio threshold on inferred fitting. (A) Effect of relative subpopulation size and sugar utilization to accuracy of inferred fit. Using computationally pooled data, the relative subpopulation sugar utilization (x-axis) and size (y-axis) were varied and the error in the inferred number of states (top), subpopulation size (middle) and sugar utilization (bottom) were calculated. In all heat maps, blue represents the highest accuracy and red the lowest. For subpopulation size and sugar utilization, only fits predicted to be two-state were used; black indicates a lack of two-state fits for that combination. Error was calculated as the average absolute deviation between the actual and predicted values. (B) Contribution of *f*-ratio threshold to accuracy of inferred fit. Computationally pooled data was analyzed using a range of *f*-ratio thresholds. Increasing the stringency of the threshold decreased the percent of two-state populations identified (top), but also decreased the error in inferred subpopulation size (middle) and sugar usage (bottom). Blue boxes represent the interquartile range with a red line at the median. Outliers (>1.5x the interquartile range) are shown as a blue x.
(EPS)

S3 Fig Shifting cells between carbon sources does not increase metabolic recycling. (A) Decay of the initial sugar after a carbon shift is plotted by the number of doublings, with the gray line indicating the expected decrease based on growth mediated dilution. Points correspond to the mean value of 29 amino acid fragments, with error bars representing the standard error of the mean. Samples shifted from ^13^C to ^12^C sugars are shown at left, and those shifted from ^12^C to ^13^C are shown at right. (B) The relative mass distribution of leucine fragment 3, proline fragment 3, and tyrosine fragment 1 before and after each carbon shift, separated into pure ^12^C fragments (blue), pure ^13^C fragments (yellow), and mixed species, which include all intermediates (green). The average percentage of intermediate species present in the 29 intermediate amino acid species after each shift is listed at right ± the standard error of the mean.
(EPS)

S1 Table Amino acid fragments used for analysis. All fragments with signal from GC-MS are listed. The 29 fragments used for the fitting analysis were selected for ease of modeling, reproducibility and lack of interfering species. The subset of 8 fragments selected for analysis of intermediate mass species in the switching experiments were selected for synthesis from multiple carbon sources and lack of anaplerotic synthesis (incorporation of CO_2_ from the air).
(XLS)

S2 Table Raw amino acid fragment mass distributions derived from FiatFlux before correction. (XLS)

S3 Table Experimental sample descriptions and CDF filenames with fit data. Samples were fit using one and two state models, and f-values, population fractions, sugar utilization characteristics and biochemical parameters (see Fig 1 for abbreviations) are reported below.
(XLS)

S4 Table Fitting data from computationally pooled samples. Samples were fit using one and two state models, and f-values, population fractions, sugar utilization characteristics and biochemical parameters (see Fig 1 for abbreviations) are reported below. In pooled sample name, MG is a mix then grow sample, and GM is a grow then mix sample. The ratios appended to the MG/GM abbreviation are the ^12^C:^13^C ratio of the experimental samples used, and the following ratio describes the computational pooling ratio between the two samples. All three replicates were fit. For information about the experimental samples, see S2 Table.
(XLS)

S1 Text Annotated MATLAB code indicating the biochemical pathways and reactions used in the amino acid model. Full set of code and functions has been uploaded to the Dryad Digital Repository (doi:10.5061/dryad.gf80t).
(PDF)

## References

[1] Raj A, van Oudenaarden A. Nature, Nurture, or Chance: Stochastic Gene Expression and Its Consequences. Cell. 2008; 135(2): 216–226. 10.1016/j.cell.2008.09.050 18957198 PMC3118044

[2] Bigger JW. Treatment of staphylococcal infections with penicillin. Lancet. 1944; 244(6320): 497–500. 10.1016/S0140-6736(00)74210-3

[3] Balaban NQ, Merrin J, Chait R, Kowalik L, Leibler S. Bacterial persistence as a phenotypic switch. Science. 2004; 305: 1622–1625. 10.1126/science.1099390 15308767

[4] Buettner F, Natarajan KN, Casale FP, Proserpio V, Scialdone A, Theis FJ, et al Computational analysis of cell-to-cell heterogeneity in single-cell RNA-sequencing data reveals hidden subpopulations of cells. Nat Biotechnol. 2015; 33: 155–60. 10.1038/nbt.3102 25599176

[5] Macosko EZ, Basu A, Satija R, Nemesh J, Shekhar K, Goldman M, et al Highly Parallel Genome-wide Expression Profiling of Individual Cells Using Nanoliter Droplets. Cell. 2015; 161: 1202–1214. 10.1016/j.cell.2015.05.002 26000488 PMC4481139

[6] Klein AM, Mazutis L, Akartuna I, Tallapragada N, Veres A, Li V, et al Droplet Barcoding for Single-Cell Transcriptomics Applied to Embryonic Stem Cells. Cell. 2015; 161: 1187–1201. 10.1016/j.cell.2015.04.044 26000487 PMC4441768

[7] Justus CR, Sanderlin EJ, Yang L V. Molecular connections between cancer cell metabolism and the tumor microenvironment. International Journal of Molecular Sciences. 2015; 16(5): 11055–11086. 10.3390/ijms160511055 25988385 PMC4463690

[8] Ahlqvist KJ, Suomalainen A, Hämäläinen RH. Stem cells, mitochondria and aging. Biochimica et Biophysica Acta—Bioenergetics. 2015; 1847(11): 1380–1386. 10.1016/j.bbabio.2015.05.014 26014347

[9] van Dijk G, van Heijningen S, Reijne AC, Nyakas C, van der Zee EA, Eisel ULM. Integrative neurobiology of metabolic diseases, neuroinflammation, and neurodegeneration. Front Neurosci. 2015; 9: 173 10.3389/fnins.2015.00173 26041981 PMC4434977

[10] Heinemann M, Zenobi R. Single cell metabolomics. Current Opinion in Biotechnology. 2011; 22(1): 26–31. 10.1016/j.copbio.2010.09.008 20934866

[11] Vasdekis AE, Stephanopoulos G. Review of methods to probe single cell metabolism and bioenergetics. Metabolic Engineering. 2015; 27: 115–135. 10.1016/j.ymben.2014.09.007 25448400 PMC4399830

[12] Ghosh A, Nilmeier J, Weaver D, Adams PD, Keasling JD, Mukhopadhyay A, et al A peptide-based method for 13C Metabolic Flux Analysis in microbial communities. PLoS Comput Biol. 2014; 10: e1003827 10.1371/journal.pcbi.1003827 25188426 PMC4154649

[13] Gebreselassie NA, Antoniewicz MR. (13)C-metabolic flux analysis of co-cultures: A novel approach. Metab Eng. 2015; 31: 132–9. 10.1016/j.ymben.2015.07.005 26219674 PMC5897767

[14] Christensen B, Nielsen J. Isotopomer analysis using GC-MS. Metab Eng. 1999; 1: 282–290. 10.1006/mben.1999.0117 10937821

[15] Szyperski T. Biosynthetically directed fractional 13C-labeling of proteinogenic amino acids. An efficient analytical tool to investigate intermediary metabolism. Eur J Biochem. 1995; 232: 433–448. 7556192 10.1111/j.1432-1033.1995.tb20829.x

[16] Nargund S, Misra A, Zhang X, Coleman GD, Sriram G. Flux and reflux: metabolite reflux in plant suspension cells and its implications for isotope-assisted metabolic flux analysis. Mol Biosyst. 2014; 10: 1496–508. 10.1039/c3mb70348g 24675729

[17] Zamboni N, Fendt S-M, Rühl M, Sauer U. 13C-based metabolic flux analysis. Nat Protoc. 2009; 4: 878–892. 10.1038/nprot.2009.58 19478804

[18] Wiechert W, Möllney M, Petersen S, de Graaf AA. A Universal Framework for 13C Metabolic Flux Analysis. Metab Eng. 2001; 3: 265–283. 10.1006/mben.2001.0188 11461148

[19] Akaike H. A new look at the statistical model identification. IEEE Trans Autom Control. 1974; 19: 716–723. 10.1109/TAC.1974.1100705

[20] Schwarz G. Estimating the dimension of a model. Ann Stat. 1978; 6: 461–464. 10.1214/aos/1176344136

[21] Canelas AB, Van Gulik WM, Heijnen JJ. Determination of the cytosolic free NAD/NADH ratio in Saccharomyces cerevisiae under steady-state and highly dynamic conditions. Biotechnol Bioeng. 2008; 100: 734–743. 10.1002/bit.21813 18383140

[22] Wang J, Atolia E, Hua B, Savir Y, Escalante-Chong R, Springer M. Natural Variation in Preparation for Nutrient Depletion Reveals a Cost-Benefit Tradeoff. PLoS Biol. 2015; 13(1): e1002041 10.1371/journal.pbio.1002041 25626068 PMC4308108

[23] Venturelli OS, Zuleta I, Murray RM, El-Samad H. Population Diversification in a Yeast Metabolic Program Promotes Anticipation of Environmental Shifts. PLoS Biol. 2015; 13(1): e1002042 10.1371/journal.pbio.1002042 25626086 PMC4307983

[24] Escalante-Chong R, Savir Y, Carroll SM, Ingraham JB, Wang J, Marx CJ, et al Galactose metabolic genes in yeast respond to a ratio of galactose and glucose. Proc Natl Acad Sci U S A. 2015; 112: 1636–41. 10.1073/pnas.1418058112 25605920 PMC4321281

[25] Nurse P, Wiemken A. Amino acid pools and metabolism during the cell division cycle of arginine-grown Candida utilis. J Bacteriol. 1974; 117: 1108–1116. Available: http://www.ncbi.nlm.nih.gov/pubmed/4591945. 4591945 10.1128/jb.117.3.1108-1116.1974 PMC246590

[26] Kotte O, Volkmer B, Radzikowski JL, Heinemann M. Phenotypic bistability in Escherichia coli’s central carbon metabolism. Mol Syst Biol. 2014; 10: 736 10.15252/msb.20135022 24987115 PMC4299493

[27] van Heerden JH, Wortel MT, Bruggeman FJ, Heijnen JJ, Bollen YJM, Planqué R, et al Lost in Transition: Startup of Glycolysis Yields Subpopulations of Nongrowing Cells. Science. 2014; 343(6174): 1245114 2014;343: 1245114–1245114. 10.1126/science.1245114 24436182

[28] Ozbudak EM, Thattai M, Lim HN, Shraiman BI, Van Oudenaarden A. Multistability in the lactose utilization network of Escherichia coli. Nature. 2004; 427: 737–740. 10.1038/nature02298 14973486

[29] Acar M, Becskei A, van Oudenaarden A. Enhancement of cellular memory by reducing stochastic transitions. Nature. 2005; 435: 228–232. 15889097 10.1038/nature03524

[30] Seth EC, Taga ME. Nutrient cross-feeding in the microbial world. Frontiers in Microbiology. 2014; 5:350 10.3389/fmicb.2014.00350 25071756 PMC4086397

[31] Mee MT, Collins JJ, Church GM, Wang HH. Syntrophic exchange in synthetic microbial communities. Proc Natl Acad Sci U S A. 2014; 111: E2149–56. 10.1073/pnas.1405641111 24778240 PMC4034247

[32] Shou W, Ram S, Vilar JMG. Synthetic cooperation in engineered yeast populations. Proc Natl Acad Sci U S A. 2007; 104: 1877–82. 10.1073/pnas.0610575104 17267602 PMC1794266

[33] Zamboni N. 13C metabolic flux analysis in complex systems. Current Opinion in Biotechnology. 2011; 22(1): 103–108. 10.1016/j.copbio.2010.08.009 20833526

