# Supplementary material for: Identifying Metabolic Subpopulations from Population Level Mass Spectrometry
Source: PLoS One. 2016 Mar 17;11(3):e0151659. doi: 10.1371/journal.pone.0151659 (PMC4795775; doi:10.1371/journal.pone.0151659)
Supplement: S1 Text — Full set of code and functions has been uploaded to the Dryad Digital Repository (doi:10.5061/dryad.gf80t). (PDF) [file pone.0151659.s008.pdf]

S1 Text. Annotated MATLAB code indicating the biochemical pathways and reactions used in the amino acid model. Full set of code and functions has been uploaded to the Dryad Digital Repository.

```
%% GLYCOLYSIS AND PENTOSE PHOSPHATE PATHWAYS

% Initialization of model structure
M = struct();

% Glucose
M.GLC(1).name = 'All';
M.GLC(1).fraction = '1';
M.GLC(1).Cn = [1 2 3 4 5 6]; %[C1 C2 C3 C4 C5 C6]
M.GLC(1).C = [1 1 1 1 1 1]; %[C1 C2 C3 C4 C5 C6]
M.GLC(1).CO2 = [0 0 0 0 0 0];
M.GLC(1).origin = 'GLU_123456';

% CO2
M.CO2(1).name = 'All';
M.CO2(1).fraction = '1';
M.CO2(1).Cn = [7];
M.CO2(1).C = [1];
M.CO2(1).CO2 = [1];
M.CO2(1).origin = 'CO2_7';

% Glucose -> Glucose-6-phosphate -> Fructose-6-phosphate
M = calcRxn(M,'glycolysis','1',{'F6P'},{{'GLC',1:6}}); % derived from a
single glucose

% Fructose-6-phosphate -> GAP + DHAP
M = calcRxn(M,'glycolysis','0.5',{'GAP','DHAP'},{{'F6P',4:6},{'F6P',3:-
1:1}});

% DHAP -> GAP
M = calcRxn(M,'glycolysis','',{'GAP'},{{'DHAP',1:3}});

% GAP -> 3-Phosphoglycerate (P3G)
M = calcRxn(M,'glycolysis','',{'P3G'},{{'GAP',1:3}});

% P3G -> Phosphoenolpyruvate (PEP)
M = calcRxn(M,'glycolysis','',{'PEP'},{{'P3G',1:3}});

% PEP -> Pyruvate
M = calcRxn(M,'glycolysis','',{'PYR'},{{'PEP',1:3}});

% Pyruvate -> Acetyl-CoA
M = calcRxn(M,'glycolysis','',{'AcCoA'},{{'PYR',2:3}});

% Erythrose-4-phosphate
M.E4P(1).name = 'PPP';
M.E4P(1).fraction = '(GVP)';
M.E4P(1).Cn = [9 9 9 9];
M.E4P(1).C = [1 1 1 1];
M.E4P(1).CO2 = [0 0 0 0];
```

```
M.E4P(1).origin = 'E4P';

M.E4P(2).name = 'PPP';
M.E4P(2).fraction = '(1-GVP)';
M.E4P(2).Cn = [9 9 9 9];
M.E4P(2).C = [1 2 2 2];
M.E4P(2).CO2 = [0 0 0 0];
M.E4P(2).origin = 'E4P';

%% TCA CYCLE

% --- AP0 cycle (entry into TCA from anapleurotic synthesis) ---

% Anapleurotic synthesis (AP0): Pyruvate + CO2 -> Oxaloacetate
M = calcRxn(M,'AP0','',{'OXA'},{{'PYR',1:3;'CO2',1}});

% Calculate the amount of AP0 OXA symmetrized by reversal of the TCA cycle
% to fumarate (fraction flipped = FS, fraction not flipped = (1-FS), where
% FS <= 0.5).
M = fumSymRxn(M,'OXA','AP0');

% AP0: Oxaloacetate + Acetyl-CoA -> Isocitrate (C order = [O4 O3 O2 A2 A1
O1])
M = calcRxn(M,'AP0','',{'ISOC'},{{'OXA', 4:-1:1; 'AcCoA', 2:-
1:1}},'rearrange',[1 2 3 5 6 4]);

% AP0 / TCA: Isocitrate ->-> a-Ketoglutarate + CO2
M = calcRxn(M,'AP0','',{'AKG'},{{'ISOC',1:5}});

% AP0 / TCA: a-Ketoglutarate ->-> Succinate + CO2
M = calcRxn(M,'AP0','',{'SUC'},{{'AKG',2:5}});

% AP0 / TCA: symmetrization at SUC/FUM stage - forward results in 50/50 of
% each orientation
M = fumSymRxn(M,'SUC','AP0');

% AP0 / TCA: Succinate ->-> Malate
M = calcRxn(M,'AP0','',{'MAL'},{{'SUC',1:4}});


% --- AP1 cycle (1 cycle after anaplerotic synthesis ---

% AP1 / TCA: Malate -> Oxaloacetate
M = calcRxn(M,'AP1','',{'OXA'},{{'MAL',1:4}});

% AP1: Oxaloacetate + Acetyl-CoA -> Isocitrate (C order = [O4 O3 O2 A2 A1
O1])
M = calcRxn(M,'AP1','',{'ISOC'},{{'OXA', 4:-1:1; 'AcCoA', 2:-
1:1}},'rearrange',[1 2 3 5 6 4],'subset',{'OXA','AP1'});

% AP1 / TCA: Isocitrate ->-> a-Ketoglutarate + CO2
M = calcRxn(M,'AP1','',{'AKG'},{{'ISOC',1:5}},'subset',{'ISOC','AP1'});

% AP1 / TCA: a-Ketoglutarate ->-> Succinate + CO2
```

```
M = calcRxn(M,'AP1','',{'SUC'},{{'AKG',2:5}},'subset',{'AKG','AP1'});

% AP1 / TCA: symmetrization at SUC/FUM stage - forward results in 50/50 of
% each orientation
M = fumSymRxn(M,'SUC','AP1');

% AP1 / TCA: Succinate ->-> Malate
M = calcRxn(M,'AP1','',{'MAL'},{{'SUC',1:4}},'subset',{'SUC','AP1'});


% --- AP2 cycle (2+ cycles after anaplerotic synthesis - all future cycles
converge)---
% AP2 / TCA: Malate -> Oxaloacetate
M = calcRxn(M,'AP2','',{'OXA'},{{'MAL',1:4}},'subset',{'MAL','AP1'});

% AP2: Oxaloacetate + Acetyl-CoA -> Isocitrate (C order = [O4 O3 O2 A2 A1
O1])
M = calcRxn(M,'AP2','',{'ISOC'},{{'OXA', 4:-1:1; 'AcCoA', 2:-
1:1}},'rearrange',[1 2 3 5 6 4],'subset',{'OXA','AP2'});

% AP2 / TCA: Isocitrate ->-> a-Ketoglutarate + CO2
M = calcRxn(M,'AP2','',{'AKG'},{{'ISOC',1:5}},'subset',{'ISOC','AP2'});

% AP2 / TCA: a-Ketoglutarate ->-> Succinate + CO2
M = calcRxn(M,'AP2/TCA','',{'SUC'},{{'AKG',2:5}},'subset',{'AKG','AP2'});

% AP2 / TCA: symmetrization at SUC/FUM stage - forward results in 50/50 of
% each orientation
M = fumSymRxn(M,'SUC','AP2');

% AP2 / TCA: Succinate ->-> Malate
M = calcRxn(M,'AP2','',{'MAL'},{{'SUC',1:4}},'subset',{'SUC','AP2'});

% --- APPEND AP FACTORS TO FRACTIONS ---
% For the set of metabolites formed during each cycle, append a term to fit
% the total proportion of that metabolite, based on the amount of
% anaplerotic synthesis versus TCA cycling (AP).  For AP0 = AP, AP1 =
% AP*(1-AP), AP2 = (1-AP)^2.

metabolites = fieldnames(M);

categories = {'AP0','AP1','AP2'};
appendFraction = {'AP*(','AP*(1-AP)*(','(1-AP)^2*('};

for i = 1:length(metabolites)

    metabolite = metabolites{i};

    for j = 1:length(M.(metabolite))
        matchName = find(strcmp(categories,M.(metabolite)(j).name(1:3)));
        if ~isempty(matchName)
            M.(metabolite)(j).fraction = [appendFraction{matchName}
M.(metabolite)(j).fraction ')'];
        end
```

```matlab
    end
end

clear i j categories appendFraction matches matchLength matchString matchName
metabolite metabolites

%% AMINO ACID SYNTHESIS
%  Calculate the composition of each amino acid based on its metabolite
%  precursor composition in structure M_AA

AA = M;

% ALANINE
AA = calcRxn(AA,'AA','',{'ALA'},{{'PYR',1:3}});

% ASPARTIC ACID
AA = calcRxn(AA,'AA','',{'ASP'},{{'OXA',1:4}});

% GLUTAMIC ACID
AA = calcRxn(AA,'AA','',{'GLU'},{{'AKG',1:5}});

% LEUCINE
AA =
calcRxn(AA,'AA','',{'LEU'},{{'AcCoA',1:2;'PYR',2:3;'PYR',2:3}},'rearrange',[1
2 3 5 6 4]);

% LYSINE
AA = calcRxn(AA,'AA','',{'LYS'},{{'AcCoA',1:2;'AKG',2:5}});

% PHENYLALANINE
AA = calcRxn(AA,'AA','',{'PHE'},{{'PEP',1:3;'PEP',2:3;'E4P',4:-1:1}});

% PROLINE
AA = calcRxn(AA,'AA','',{'PRO'},{{'GLU',1:5}});

% THREONINE
AA = calcRxn(AA,'AA','',{'THR'},{{'ASP',1:4}});

% TYROSINE
AA = calcRxn(AA,'AA','',{'TYR'},{{'PEP',1:3;'PEP',2:3;'E4P',4:-1:1}});

% VALINE
AA = calcRxn(AA,'AA','',{'VAL'},{{'PYR',1:3;'PYR',2:3}},'rearrange',[1 2 4 5
3]);

% ISOLEUCINE
AA = calcRxn(AA,'AA','',{'ILE'},{{'THR',1:4;'PYR',2:3}},'rearrange',[1 2 5 6
3 4]);

% GLYCINE
AA = calcRxn(AA,'AA','(1-SVT)',{'GLY'},{{'THR',1:2}});
AA = calcRxn(AA,'AA','SVT*(1-THF)',{'GLY'},{{'P3G',1:2}});
AA = calcRxn(AA,'AA','SVT*(1-SVT)*(THF)',{'GLY'},{{'THR',1:2}});
AA = calcRxn(AA,'AA','SVT^2*(THF)',{'GLY'},{{'P3G',1:2}});
```

```
% SERINE
AA = calcRxn(AA,'AA_No_THF','(1-THF)',{'SER'},{{'P3G',1:3}});
AA = calcRxn(AA,'AA_THF','THF',{'SER'},{{'GLY',1:2;'GLY',1}});

% Create M_AA structure containing only the amino acids
M_AA = struct();

AAlist =
{'ALA','ASP','GLU','GLY','ILE','LEU','LYS','PHE','PRO','SER','THR','TYR','VAL
'};
for i = 1:length(AAlist)
    M_AA.(AAlist{i}) = AA.(AAlist{i});
end
clear i AAlist
```
